# Suicidal Ideation, Attempt, and Determining Factors among HIV/AIDS Patients, Ethiopia

**DOI:** 10.1155/2016/8913160

**Published:** 2016-09-22

**Authors:** Huluagresh Bitew, Gashaw Andargie, Agitu Tadesse, Amsalu Belete, Wubalem Fekadu, Tesfa Mekonen

**Affiliations:** ^1^Debark District Hospital Northwest, Debark, Ethiopia; ^2^School of Public Health, College of Medicine and Health Science, University of Gondar, Gondar, Ethiopia; ^3^Amanuel Mental Specialized Hospital, Addis Ababa, Ethiopia; ^4^College of Health Science, Debre Tabor University, Debre Tabor, Ethiopia; ^5^Psychiatry Department, College of Medicine and Health Sciences, Bahir Dar University, Bahir Dar, Ethiopia

## Abstract

*Background*. Suicide is a serious cause of mortality worldwide and is considered as a psychiatric emergency. Suicide is more frequent in peoples living with HIV/AIDS than in general population.* Objective*. To assess the proportion and determining factors of suicidal ideation and attempt among peoples living with HIV/AIDS in Ethiopia.* Methods*. Institutional based cross-sectional study was conducted from May to June 2015 by selecting 393 participants using systematic random sampling technique. Suicide manual of Composite International Diagnostic Interview (CIDI) was used to collect data. Logistic regression was carried out and odds ratio with 95% confidence intervals was computed.* Results*. The proportion of suicidal ideation and attempt was 33.6% and 20.1%, respectively. Female sex (AOR = 2.6, 95%CI: 1.27–5.22), marital status (AOR = 13.5, 95%CI: 4.69–39.13), depression (AOR = 17.0, 95%CI: 8.76–33.26), CD4 level (AOR = 2.57, 95%CI: 1.34–4.90), and presence of opportunistic infection (AOR = 5.23, 95%CI: 2.51–10.88) were associated with suicidal ideation, whereas marital status (AOR = 8.44, 95%CI: 3.117–22.84), perceived HIV stigma (AOR = 2.9, 95%CI: 1.45–5.99), opportunistic infection (AOR = 2.37, 95%CI: 1.18–4.76), and poor social support (AOR = 2.9, 95%CI: 1.58–5.41) were significantly associated with suicidal attempt.* Conclusion*. Suicidal ideation and attempt were high among HIV positive patients. Therefore early screening, treatment, and referral of suicidal patients are necessary in HIV clinics.

## 1. Background

Suicide can be defined as intentional self-inflicted death. Suicide attempt is an intentional but unsuccessful act of killing one self and suicidal ideation is thought about killing oneself [1]. According to WHO (World Health Organization), a person commits suicide in every 40 seconds somewhere in the world and every 3 seconds, a person attempts to die. A single suicide has a serious psychological, social, and financial impact on at least six other people [2]. Suicide accounts 1% of the disease burden in the world [3].

HIV/AIDS is a common health care issue and currently more than 34 million people live with HIV/AIDS globally [4] and 23.5 million (69%) of them live in sub-Saharan Africa. It is a significant cause of death and disability, especially in low- and middle-income countries [5, 6].

Mental health and HIV/AIDS are closely interlinked; mental health problems increased risk of having HIV/AIDS and interfere with its treatment due to lack of insight about the illness and decision-making problems, and conversely some mental disorders occur as a direct result of HIV infection which may result from stigma, opportunistic infections, or medication side effect [7]. Despite advances in therapy that transformed HIV/AIDS into a treatable chronic illness, it continues to be associated with an underrecognized risk for suicidal ideation as well as attempted and completed suicide [8]. Compared to the general population, the risk of completed suicide occurs 7 to 36 times greater in people with HIV/AIDS [9].

Different studies revealed that prevalence of suicidal ideation among peoples with HIV/AIDS was 52% in Australia and 31% in London while suicidal attempt was 23% in France [10–12]. A cross-sectional study conducted in semiurban Uganda among 618 attendants at two HIV clinics reveals the prevalence of moderate to high risk for suicide as 7.8% and life time attempted suicide of 3.9% [13]. Another survey in University of Benin Teaching Hospital among 150 HIV positive individuals reported that suicidal ideation was 43% [14]. In Ethiopia, population based study shows that the prevalence of suicidal ideation and suicidal attempt was 2.7% and 0.9%, respectively [15].

Suicide was significantly associated with perceived social support, perceived HIV stigma, major depression, and higher education in USA [16]. In France suicide was associated with female sex, younger age, being HIV positive, HIV-discrimination, and the lack of social support [12]. Gender violence in Brazil, discrimination and depression in India, early infection period in New York were significantly associated with suicidal ideation among peoples with HIV/AIDS [16–18]. In South Africa a partner relational problems and mental and medical conditions were significantly associated with suicidal behavior [19]. However little is known about suicidal behavior in Ethiopian HIV clinics and this study aimed to assess the proportion and determining factors of suicidal ideation and attempt among people living with HIV/AIDS, Northwest, Ethiopia, 2015.

## 2. Materials and Methods

Institution based cross-sectional study was conducted from April to May 2015 among people with HIV/AIDS at Debark District Hospital Northwest, Ethiopia. A town 836 KM far from the capital Addis Ababa. The sample required for the study was 393 which was determined using single population proportion formula considering proportion of suicide as 42% in Benin [14], 5% margin of error, 95% confidence level, and 10% nonresponse rate. Systematic random sampling method was used to select participants from a total of 2340 patients coming to Debark Hospital during the study period.

Presence or absence of suicidal ideation and suicidal attempt was the dependent variable whereas sociodemographic variables, wealth index, clinical factors (CD4 count, HIV/AIDS stage, and opportunistic infection), psychosocial factors, stigma, substance use variables, and social support were the independent variables.

Data were collected by interviewing the selected participants and reviewing medical records. WHO's Composite International Diagnostic Interview (CIDI) suicide manual was used to assess suicidal ideation and attempt [20]. Depression was assessed by Patients Health Questionnaire version-9 (PHQ 9) [21] while social support was measured using Oslo Social Support scale [22] and stigma related to HIV/AIDS was assessed by perceived HIV Stigma scale [23].

Data was entered to Epi info version 7 and exported to SPSS-20 for further analysis. Descriptive statistic was used to explain the study participants in relation to study variables. Multiple logistic regression analysis was conducted to identify determining factors of suicidal ideation and attempt with persons living with HIV/AIDS. The strength of the association was presented by odds ratio with 95% confidence interval and *P* value less than 0.05 was considered significant.

## 3. Results

### 3.1. Sociodemographic Characteristics

A total of 393 individuals participated in the study with mean age of 37 ± 9.15. Two hundred thirty (58.5%) of the respondents were female, 255 (64.9%) of the participants were unemployed, 127 (32.3%) of the respondents were single, 63 (16%) were in the lowest socioeconomic status of wealth index, and 80 (20.4%) of the respondents live alone (Table 1).

### 3.2. Clinical Characteristics

Three hundred fifty-two (89.6%) of the respondents were diagnosed for HIV before 6 months of data collection time, 269 (68.4%) of respondents had a CD4 level greater than 500, and 319 (81.2%) of them were on the first WHO stages of HIV/AIDS. Most of the respondents (93.9%) were on HAART (Highly Active Antiretroviral Therapy).

Two hundred (50.9%) of the respondents know their partners serum status and 200 (50.9%) of the participants had no OI. One hundred forty-nine (37.9%) of the respondents had depression (score > 5 in PHQ-9) and 115 (29.3%) of respondents perceive as other people stigmatize them because they are positive for HIV (Table 2).

### 3.3. Substance Use

Three hundred eight (77.1%) of the respondents had history of alcohol use at least once in their life, 28 (7.1%) of them were chewing khat (*Catha edulis*, a local amphetamine like substance), 17 (4.3%) of the respondents reported that they smoke tobacco, and 151 (38.4%) of the participants were currently drinking alcohol for nonmedical purpose (Table 3).

#### 3.3.1. Suicidal Ideation and Attempt

One hounded thirty-two (33.6%) of the respondents had suicidal ideation and of them, 22 (23.4%) reported that they had suicidal ideation within 6 months after they knew their serum status. Life time suicidal attempt was 20.1% in the participants. From the total participants, 1.8% had suicidal attempt in the last one month whereas 13 (2.4%) of the respondents attempted suicide within 3 months after knowing their serum status.

Regarding the frequency of suicidal attempt 29 (50.0%), 21 (36.2%), and 8 (13.8%) of respondents attempted once, twice, and more than two times in their life time, respectively (Table 4).

### 3.4. Determining Factors of Suicidal Ideation

In multivariate logistic regression, suicidal ideation was significantly associated with being single, female sex, having CD4 level <500, presence of OI, depression, and poor social support (Table 5).

### 3.5. Determining Factors of Suicidal Attempt

The results of multivariate analysis showed that suicidal attempts were significantly associated with being single, having OI, presence of perceived stigma, and poor social support. On the other hand, sex had not significantly associated with suicidal attempt on multivariate logistic regression (Table 6).

## 4. Discussion

This study confirms that suicidal ideation is 33.6% among HIV positive patients, which was lower than studies in Benin 43% [14], Australia 52% [10], and Ilorin 56.7% [13] whereas it is in line with a study conducted in London 31% [11]. On the other hand suicidal ideation was higher than studies conducted among HIV positive population at semiurban setting of Nigeria (7.6%) [24]. In this study 20.1% of the respondents were exposed to suicidal attempt, which is higher than community based studies conducted in Addis Ababa 0.9% [15] and Uganda 3.9% [13]. On the other hand, the study revealed that suicidal attempt among HIV positive patients was in line with other studies in France 23% [12].

Sex was significantly associated with suicidal ideation. Females are 2.3 times more likely to have suicidal ideation than male patients. This is supported by previous studies where ideation is higher in females where completed suicide is higher among males [12, 14, 17]. Marital status was also found to be significantly associated with suicidal ideation. Patients who were single or living alone were 13.5 times more likely to develop suicidal ideation than patients who were married, which is supported by other studies [15, 25].

Patients who have OI were two times more likely to attempt suicide as compared with their counterparts. This may be due to being physically weak and emaciated which results in hopelessness and end up with suicide attempt. Other similar studies also support the result [2, 26].

Social support was significantly associated with suicidal ideation. This can be explained as where support is available alternative options may be available before a person decides to think about death. This is also supported by other similar studies conducted in France, New York, and South Africa [12, 16, 19]. Patients with CD4 level <500 were 2.5 times more likely to have suicidal ideation than with CD4 level >500. OI and depression were found to be significantly associated with suicidal ideation. This can be due to physical complications or fear of death.

Concerning associated factors of suicidal attempt, HIV positive patients who were stigmatized had been exposed 3 times to suicidal attempt compared to those who were not being stigmatized. This may be due to the fact that feeling of stigmatized might contribute frequent psychological stress and finally lead to suicidal attempt. This is in agreement with other studies [17].

Marital status was found to be significantly associated with suicidal attempt. HIV positive patients who are single were 8.4 times more likely to be exposed to suicidal attempt than married HIV positive patients. Being alone leads to repeated stressful life and also this burden disturbs the social and emotional functioning, finally contributed for suicidal attempt. Other studies support this result [25].

This study also confirms that social support was another factor for suicidal attempt. Patients who had poor social support were 2.2 times more likely to experience suicidal attempt than those who were in good social support. The possible reason might be that those HIV positive patients who were in poor social support may think as they are alone and it increases stress and leads to suicidal attempt. This is also supported by other similar studies [26].

## 5. Conclusion and Recommendations

Suicidal ideation and attempt are high among HIV/AIDS patients and the contributing factors are being female, being single, presence of opportunistic infection, depression, stigma, and having poor social support. So screening, treatment, and referral service of suicidal individuals in HIV clinics are essential. Moreover, clinicians need to give more attention to people with comorbid depression and having poor social support. Finally by modifying some factors like opportunistic infection and depression we may reduce suicidal behavior.

## Figures and Tables

**Table 1:** Sociodemographic characteristics of HIV patients at Debark Hospital Northwest, Ethiopia, 2015 (*n* = 393).

Variable	Category	Frequency (*n*)	Percentage (%)
Age in year	18–27	37	9.4
	28–37	150	38.2
	38–47	132	33.6
	48+	74	18.8

Sex	Male	163	41.5
	Female	230	58.5

Ethnicity	Amhara	388	98.7
	Tigre	5	1.3

Marital status	Single	127	32.3
	Widowed	46	11.7
	Divorced	118	30
	Married	102	26

Religion	Orthodox	371	94.4
	Muslim	22	5.6

Educational status	Not literate	157	39.9
	Primary	93	23.7
	Secondary	102	25.9
	Higher education	44	11.2

Occupation	Unemployed	255	64.9
	Employed	138	35.1

Living condition	With family	313	79.6
	Alone	80	20.4

Wealth index	Lowest	63	16
	Second	50	12.7
	Medium	205	52.2
	Fourth	9	2.3
	Highest	66	16.8

Social support	Poor	146	37.2
	Intermediate	208	52.9
	Good	39	9.9

**Table 2:** Clinical characteristics of HIV positive patients at Debark Hospital Northwest, Ethiopia, 2015 (*n* = 393).

Variable	Category	Frequency (*n*)	Percent (%)
Serum status knowing duration	<6 months	41	10.4
	≥6 months	352	89.6

WHO AIDS stage	T1	319	81.2
	T2	5	1.3
	T3	1	0.3
	1	16	4.1
	2	28	7.1
	3	18	4.6
	4	6	1.5

CD4 level	≤500	124	31.6
	>500	269	68.4

Starting HAART	Yes	369	93.9
	No	24	6.1

Partner serum status	Positive	146	37.2
	Negative	54	13.7
	Not known	193	49.1

Opportunistic infections (OI)	TB	327	83.2
	Herpes zoster	62	15.8
	Others	4	1

Depression	Yes	149	37.9
	No	244	62.1

Perceived HIV stigma	Yes	115	29.3
	No	278	70.7

**Table 3:** Substance use of HIV positive respondents at Debark Hospital Northwest, Ethiopia, 2015 (*n* = 393).

Variable	Categories	Frequency (*n*)	Percentage (%)
Life time alcohol use	Yes	308	77.1
	No	85	22.9

Life time khat chewing	Yes	28	7.1
	No	365	92.9

Life time smoking	Yes	17	4.3
	No	376	95.7

Current alcohol use	Yes	151	38.4
	No	242	61.6

Current khat chewing	Yes	11	2.8
	No	382	97.2

Current smoking	Yes	8	2
	No	385	98

**Table 4:** Suicidal ideation and attempt among respondents attending ART clinic at Debark Hospital Northwest, Ethiopia, 2015.

Variable	Categories	Frequency (*n*)	Percent (%)
Suicidal ideation	Male	22	5.6
	Female	110	28

Duration of suicidal ideation	<6 months	17	4.4
	≥6 months	115	29.5

Suicidal attempt in the last 1 month	Male	2	0.5
	Female	5	1.3

Life time suicidal attempt	Male	15	3.8
	Female	64	16.3

Duration of suicidal attempt	<6 months	5	1.3
	≥6 months	74	18.8

Intentional (self-inflicted) injury	Yes	131	33.3
	No	262	66.7

**Table 5:** Factors associated with suicidal ideation of participants at Debark Hospital Northwest, Ethiopia, 2015.

Variables	Categories	Suicidal ideation		COR (95% CI)	AOR (95% CI)
		Yes	No		
Sex	Male	22	141	1	1
	Female	110	120	5.8 (3.3–9.9)	**2.6** (1.3–5.2)^*∗∗*^

Social support	Good	64	207	1	1
	Poor	68	54	2.8 (1.3–6.3)	**2.5** (1.3–4.9)^*∗∗*^

Marital status	Single	83	44	15.6 (7.6–32.2)	**13.5** (4.7–39.1)^*∗∗*^
	Widowed	8	38	1.7 (0.6–4.7)	2.26 (0.9–5.8)
	Divorced	30	88	2.8 (1.3–5.9)	**2.7** (1.3–4.7)^*∗*^
	Married	11	91	1	1

CD4	≤500	88	81	4.4 (2.8–6.9)	**2.5** (1.3–4.9)^*∗∗*^
	>500	44	180	1	1

Depression	Yes	38	159	24.1 (13.8–42.0)	**17** (8.8–33.3)^*∗∗*^
	No	20	200	1	1

Perceived stigma	Yes	82	196	1.8 (1.2–2.9)	1.9 (0.9–3.8)
	No	50	65	1	1

OI	Yes	13	55	8.3 (4.9–14.2)	**5.2** (2.5–10.9)^*∗∗∗*^
	No	45	304	1	1

^*∗*^ *P* value < 0.05, ^*∗∗*^ *P* value < 0.01, and ^*∗∗∗*^ *P* value < 0.001.

**Table 6:** Factors associated with suicidal attempt of participants at Debark Hospital Northwest, Ethiopia, 2015.

Variables	Categories	Suicidal attempt		COR (95% CI)	AOR (95% CI)
		Yes	No		
Sex	Male	15	148	1	1
	Female	64	166	3.8 (2.1–6.9)	**2.8** (1.3–6.2)^*∗*^

Marital status	Single	59	68	11.7 (5.1–27.4)	**8.4** (3.1–22.8)^*∗∗∗*^
	Widowed	1	45	3.9 (1.9–8.4)	4.5 (1.9–10.3)
	Divorced	12	106	1.5 (0.6–4.1)	1.6 (0.5–4.6)
	Married	7	95	1	1

OI	Yes	10	26	2.6 (1.3–5.5)	**2.3** (1.2–4.8)^*∗∗*^
	No	48	333	1	1

Perceived stigma	Yes	62	216	2.7 (1.3–5.7)	**2.9** (1.4–5.9)^*∗∗*^
	No	17	98	1	1

Social support	Poor	60	70	3.1 (1.6–6.2)	**3.0** (1.6–5.9)^*∗∗*^
	Intermediate	53	155	1.1 (0.5–2.5)	1.2 (0.5–2.7)
	Good	9	30	1	1

^*∗*^ *P* value < 0.05, ^*∗∗*^ *P* value < 0.01, and ^*∗∗∗*^ *P* value < 0.001.

## Abbreviations

ART: Antiretroviral Therapy
CIDI: Composite International Diagnostic Interview
CD4: Cluster of differentiation-4
OR: Odds ratio
AOR: Adjusted odds ratio
OI: Opportunistic infection.

## References

[1] Sadock B. J., Sadock V. A. (2011). *Kaplan and Sadock's Synopsis of Psychiatry: Behavioral Sciences/Clinical Psychiatry*.

[2] World Health Organization (2000). *The size of the problem. Preventing Suicide. A Resource for Primary Care Workers*.

[3] World Health Organization (2008). *Scaling Up Care for Mental, Neurological, and Substance Use Disorders*.

[4] Kim A. Y., Onofrey S., Church D. R. (2013). An epidemiologic update on hepatitis C infection in persons living with or at risk of HIV infection. *Journal of Infectious Diseases*.

[5] ILO (2012). *UNAIDS Report on the Global AIDS Epidemic; Cooperation Results Overview 2005–2013*.

[6] Hajizadehet M., Drissa S., Jody H., Arijit N., Pine A. (2014). Socioecono inequalities in HIV/AIDS prevalence in sub-Saharan African countries evidence from the Demographic Health Surveys. *International Journal for Equity in Health*.

[7] World Health Organization (2008). *HIV/AIDS and Mental Health Report by the Secretariat*.

[8] Lawrence S. T., Willig J. H., Crane H. M. (2010). Routine, self-administered, touch-screen, computer-based suicidal ideation assessment linked to automated response team notification in an HIV primary care setting. *Clinical Infectious Diseases*.

[9] New York State Department of Health AIDS Institute (2007). *Suicidality and Violence in Patients With HIV/AIDS*.

[10] Kelly B., Raphael B., Judd F. (1998). Suicidal ideation, suicide attempts, and HIV infection. *Psychosomatics*.

[11] Sherr L., Lampe F., Fisher M. (2008). Suicidal ideation in UK HIV clinic attenders. *AIDS*.

[12] Préau M., Bouhnik A.-D., Peretti-Watel P., Obadia Y., Spire B., ANRS-EN12-VESPA Group (2008). Suicide attempts among people living with HIV in France. *AIDS Care*.

[13] Kinyanda E., Hoskins S., Nakku J., Nawaz S., Patel V. (2012). The prevalence and characteristics of suicidality in HIV/AIDS as seen in an African population in Entebbe district, Uganda. *BMC Psychiatry*.

[14] Chikezie U. E., Okogbenin E. O., Ebuenyi I. D., Aweh B. E. (2013). Patterns of comorbid infections and associated suicidal ideations among individuals attending HIV/AIDS clinic in Benin City. *Epidemiology*.

[15] Kebede D., Alem A. (1999). Suicide attempts and ideation among adults in Addis Ababa, Ethiopia. *Acta Psychiatrica Scandinavica, Supplement*.

[16] Galvan F. H., Davis E. M., Banks D., Bing E. G. (2008). HIV stigma and social support among African Americans. *AIDS Patient Care and STDs*.

[17] Guimarães P. M., Passos S. R., Calvet G. A., Hökerberg Y. H., Lessa J. L., de Andrade C. A. (2014). Suicide risk and alcohol and drug abuse in outpatients with HIV infection and Chagas disease. *Revista Brasileira de Psiquiatria*.

[18] Deb S., Sun J., Strodl E. (2013). Discrimination experienced by HIV/AIDS infected persons and its associations with mental health in an Indian sample. *Stigma Research and Action*.

[19] Govender R. D., Schlebusch L. (2012). Suicidal ideation in seropositive patients seen at a South African HIV voluntary counselling and testing clinic. *African Journal of Psychiatry*.

[20] Kessler R. C., Üstün B. (2004). The World Mental Health (WMH) Survey Initiative version of the World Health Organization (WHO) Composite International Diagnostic Interview (CIDI). *International Journal of Methods in Psychiatric Research*.

[21] Gelaye B., Williams M. A., Lemma S. (2013). Validity of the patient health questionnaire-9 for depression screening and diagnosis in East Africa. *Psychiatry Research*.

[22] Abiola T., Udofia O., Zakari M. (2013). Psychometric properties of the 3-item oslo social support scale among clinical students of Bayero University Kano, Nigeria. *Malaysian Journal of Psychiatry*.

[23] Feyissa G. T., Abebe L., Girma E., Woldie M. (2012). Validation of an HIV-related stigma scale among health care providers in a resource-poor Ethiopian setting. *Journal of Multidisciplinary Healthcare*.

[24] Shittu R. O., Alabi M. K., Odeigah L. O. (2014). Suicidal ideation among depressed people living with HIV/AIDS in Nigeria, West Africa. *Open Journal of Medical Psychology*.

[25] O'Dowd M. A., Biderman D. J., McKegney F. P. (1993). Incidence of suicidality in AIDS and HIV-positive patients attending a psychiatry outpatient program. *Psychosomatics*.

[26] UNAIDS (2009). *AIDS Epidemic Update*.

